# MFGE8-primed fibroblasts reprogram the immunosuppressed microenvironment to promote diabetic wound healing

**DOI:** 10.3389/fcell.2026.1810043

**Published:** 2026-05-06

**Authors:** Ling Pan, Yejing Huang, Xinfeng Wu, Dongqing Li, Hongsheng Wang

**Affiliations:** 1 Hospital for Skin Diseases, Institute of Dermatology, Chinese Academy of Medical Sciences and Peking Union Medical College, Nanjing, Jiangsu, China; 2 Department of Mycobacterium, Jiangsu Provincial Key Laboratory of Dermatology, Hospital for Skin Diseases, Institute of Dermatology, Chinese Academy of Medical Sciences and Peking Union Medical College, Nanjing, China

**Keywords:** diabetic wound, fibroblasts, inflammation, macrophage, MFGE8

## Abstract

**Background:**

Chronic diabetic ulcers exhibit a dysregulated immune microenvironment characterized by aberrant leukocyte responses and dysfunctional fibroblast activation. Identifying molecular regulators of fibroblast-driven immune responses may provide therapeutic targets to restore effective wound repair in these chronic wounds.

**Methods:**

Bulk RNA-seq (GSE199939) and scRNA-seq (GSE165816) datasets from human DFUs were integrated to identify alterations in MFGE8 expression in wound fibroblasts. These findings were validated by immunofluorescence staining and RT-qPCR in *db/db* mouse wounds. Cytokine screening assays were performed to identify upstream regulators of MFGE8 dysregulation. The effects of MFGE8 on fibroblast immune activity were assessed by RT-qPCR, Western blotting, ELISA, and Transwell migration assays. To evaluate its therapeutic potential *in vivo*, a single-dose of MFGE8-prestimulatedd fibroblasts were locally delivered into *db/db* mouse wounds, followed by wound healing assessment, immunofluorescence staining, and transcriptomic analysis.

**Results:**

MFGE8 was markedly downregulated in fibroblasts from DFUs. TNF-α and IL-1α robustly induced MFGE8 expression in dermal fibroblasts. Recombinant MFGE8 reprogrammed fibroblasts toward a pro-inflammatory phenotype, significantly increasing the expression of IL1B, IL6, CXCL8, CXCL1, and CCL2, and enhancing macrophage migration *in vitro*. In *db/db* mice, MFGE8-prestimulated fibroblasts increased macrophage infiltration, activated inflammatory and pro-repair transcriptional programs, and accelerated wound closure.

**Conclusion:**

These findings establish MFGE8 as a key regulator of fibroblast inflammatory competence and support a cell-based immunomodulatory strategy to promote healing in chronic diabetic wounds.

## 1 Introduction

Diabetic foot ulcers (DFUs) are among the most debilitating complications of diabetes and a leading cause of non-traumatic lower-limb amputation and premature mortality (Armstrong et al., 2023; GBD, 2021 Diabetes Collaborators, 2023). Epidemiological studies indicate that 19%–34% of individuals with diabetes will develop a foot ulcer during their lifetime (Armstrong et al., 2023; GBD, 2021 Diabetes Collaborators, 2023). Recurrent ulceration is common, with up to 42% of patients developing a new ulcer within 1 year after healing and 65% within 5 years, resulting in sustained healthcare utilization and substantial economic burden (Armstrong et al., 2023). Pathologically, chronic diabetic ulcers fail to progress through the orderly phases of wound repair and instead remain trapped in a state of dysregulated inflammation, impaired angiogenesis and defective matrix remodeling (Yang et al., 2023; Bauer et al., 2025; Ghebrehiwet-Kuflom et al., 2025).

Traditionally, diabetic wounds have been regarded as lesions “stuck” in an exaggerated inflammatory phase, characterized by persistent leukocyte infiltration, excessive protease activity and matrix degradation, and therapeutic strategies have therefore largely aimed at dampening inflammation (Raja et al., 2023; Wang R. et al., 2025; Huang et al., 2025). Recent bulk and single-cell transcriptomic analyses of human DFUs, however, have challenged this simplistic hyper-inflammatory paradigm (Sawaya et al., 2020; Theocharidis et al., 2020; Theocharidis et al., 2022; Valsami et al., 2025). These studies show that gene programs linked to inflammatory responses and cell migration are dysregulated and less activated compared with acute oral and skin wounds, resulting in defective neutrophil and macrophage recruitment and ineffective inflammatory response (Sawaya et al., 2020; Theocharidis et al., 2020). Single-cell RNA sequencing further revealed a distinct subset of “healing-enriched” fibroblasts, characterized by the coordinated expression of inflammatory, chemokine, and matrix-remodeling genes, which was selectively enriched in healing ulcers but markedly depleted in non-healing lesions (Theocharidis et al., 2022). Moreover, another type of chronic wound, venous leg ulcers, has been shown in recent single-cell studies to exhibit an immunosuppressive microenvironment, characterized by dysregulated inflammatory gene expression in both structural and immune cell populations (Wang J. et al., 2025). Taken together, these findings suggest that chronic diabetic ulcers exist within an ineffective inflammatory microenvironment, characterized by blunted leukocyte recruitment and attenuated stromal inflammatory programs, rather than a simply excessive or persistent inflammatory state (Sawaya et al., 2020; Theocharidis et al., 2020; Theocharidis et al., 2022; Valsami et al., 2025). Within this framework, a central unresolved question is how productive, pro-repair inflammatory programs can be selectively re-engaged in diabetic wounds, rather than globally suppressing or indiscriminately amplifying inflammatory signaling.

Milk fat globule-EGF factor 8 (MFGE8, also known as MFG-E8) is a secreted glycoprotein that bridges phosphatidylserine on apoptotic cells to αvβ3/αvβ5 integrins on phagocytes, thereby promoting efferocytosis and preventing chronic inflammation and autoimmunity (Ma et al., 2024; Liu et al., 2023). Beyond apoptotic cell clearance, MFGE8 has been implicated as a pleiotropic regulator of inflammation, tissue repair and cancer, where it modulates macrophage polarization, restrains inflammasome activation and supports angiogenesis (Liu et al., 2023; Liu W. et al., 2025; Ma et al., 2022). Previous studies demonstrate that MFGE8 protein levels are significantly reduced in wound exudates from patients with chronic diabetic ulcers compared with those from non-diabetic chronic wounds. Moreover, the remaining MFGE8 is extensively glycated, resulting in a profound loss of phosphatidylserine-binding activity and compromised biological function (Das et al., 2016; Huang et al., 2020). Although exogenous administration of recombinant MFGE8 accelerates wound closure in diabetic mouse models, direct protein therapy faces substantial translational barriers in chronic wounds (Das et al., 2016; Uchiyama et al., 2017). The chronic wound milieu is protease-rich, with elevated matrix metalloproteinases and other degradative enzymes, compounded by persistent bacterial and fungal colonization (Cho et al., 2019; Chen et al., 2023). Recent studies have identified inflammation-associated, pro-repair fibroblast subsets in diabetic ulcers that actively orchestrate stromal–immune crosstalk (Theocharidis et al., 2022). It is unclear whether MFGE8-primed fibroblasts can function as therapeutic effectors to restore immune homeostasis, rebalance chemokine networks, and reshape the pathological diabetic wound microenvironment.

Here, we demonstrate that MFGE8 expression is markedly reduced in fibroblasts isolated from human diabetic foot ulcers and venous leg ulcers, as well as from wounds of *db/db* diabetic mice. Recombinant MFGE8 converts fibroblasts into a pro-inflammatory, chemokine-producing state with increased expression of IL1β, IL6, CXCL8, CXCL1 and CCL2 and an enhanced capacity to drive macrophage migration. Using a full-thickness excisional wound model in *db/db* mice, we demonstrate that a single local treatment of MFGE8-prestimulated fibroblasts significantly accelerate diabetic wound closure. These findings identify MFGE8 as a critical immunomodulatory mediator that restores fibroblast inflammatory competence and MFGE8-pretreated fibroblasts functionally reprograms the immunosuppressed diabetic wound niche toward a regenerative state.

## 2 Materials and methods

### 2.1 Fibroblast isolation and culture

Normal skin samples were collected from patients aged 18–60 years undergoing cosmetic surgery at the Hospital for Skin Diseases, Chinese Academy of Medical Sciences. Patients with severe systemic diseases or malignant tumors were excluded. All participants were fully informed about the use of their specimens and provided written informed consent. Surgically obtained skin tissues were rinsed 3–4 times within PBS supplemented with 1% Antibiotic-Antimycotic (Thermo Fisher, United States) and sectioned into approximately 6 × 6 × 6 mm fragments. The tissue fragments were incubated in 5 U/mL Dispase II (Roche, Switzerland) at 4 °C overnight to allow epidermal separation. Then, the remaining dermal tissue was finely minced and enzymatically digested in DMEM (Gibco, United States) containing 1.5 mg/mL Collagenase I (Worthington, United States), 1.5 mg/mL Collagenase III (Worthington, United States), and 5 μg/mL DNase I (Roche, Switzerland) at 37 °C for 3–4 h. Enzymatic activity was stopped by adding DMEM supplemented with 10% fetal bovine serum (FBS; Sigma, the United States) and 1% penicillin-streptomycin (Thermo Fisher, the United States). Dermal fibroblasts were isolated by centrifugation at 150 g for 8 min and cultured in DMEM supplemented with 10% FBS and 1% penicillin-streptomycin at 37 °C in a humidified incubator with 5% CO_2_. Fibroblasts from three different donors were pooled to generate a mixed human primary fibroblast population for subsequent experiments. The cells were maintained under the conditions described above and passaged for up to eight passages.

### 2.2 RNA isolation and quantitative reverse-transcription PCR (RT‒qPCR)

Wound tissues were rapidly frozen in liquid nitrogen and then cut into small fragments before homogenization in TRIzol reagent (Thermo Fisher, United States) using a tissue grinder (Jingxin, JXFSTPRP-24). Cultured cells were lysed directly by adding TRIzol to the plates and incubated at room temperature for 5 min. Total RNA was isolated according to the manufacturer’s instructions, resuspended in nuclease-free water, and quantified using a NanoDrop One. Reverse transcription was performed using 500 ng of total RNA with HiScript III RT SuperMix (Vazyme, China). Quantitative PCR was conducted with ChamQ Universal SYBR qPCR Master Mix (Vazyme, China). Relative gene expression was normalized to GAPDH and calculated using the 2^−ΔΔCt^ method. Primer sequences are listed in Supplementary Table 1.

### 2.3 Immunoblot

Human primary fibroblasts were stimulated with recombinant MFGE8 (1 μg/mL or 2 μg/mL; Sino Biological, United States), TNFα (50 ng/mL; R&D, United States), or IL1α (10 ng/mL; R&D, United States) for 48 h. Cells were lysed by direct addition of RIPA buffer (Beyotime, China) to culture plates and incubated for 10 min to ensure complete protein extraction. Equal amounts of protein were resolved on SDS–PAGE precast gels and transferred to PVDF membranes. Membranes were blocked with 5% non-fat milk prepared in TBST for 1 h at room temperature, followed by incubation with primary antibodies at 4 °C overnight. After three washes with TBST, membranes were incubated with HRP-conjugated secondary antibodies (Thermo Fisher, United States) for 1 h at room temperature. Protein signals were developed using an ECL detection kit and captured with a chemiluminescence imaging system. Primary antibodies and second antibodies were used are listed in Supplementary Table 2.

### 2.4 Immunofluorescence

Paraffin-embedded tissue sections were deparaffinized in xylene and sequentially rehydrated using graded ethanol solutions. Antigen retrieval was carried out in Tris-EDTA buffer (pH = 9.0) at 98 °C for 30 min. Sections were permeabilized with 0.1% Triton X-100 for 20 min, followed by blocking in 5% bovine serum albumin (Sigma, United States) at room temperature for 1 h. The samples were then incubated with primary antibodies at 4 °C overnight. After three washes with TBST, fluorophore-conjugated secondary antibodies (Thermo Fisher, United States) were applied for 45 min in the dark. Finally, sections were mounted using antifade mounting medium containing DAPI (Thermo Fisher, United States) and visualized with a fluorescence microscope (Olympus, Japan). Primary antibodies and second antibodies were used are listed in Supplementary Table 2.

### 2.5 ELISA

Human primary fibroblasts were stimulated with recombinant MFGE8 (1 μg/mL; Sino Biological, United States), TNFα (50 ng/mL; R&D, United States), or IL1α (10 ng/mL; R&D, United States) for 48 h. Culture supernatants were then collected, and the levels of IL-1β, CXCL1, CCL2, and MFGE8 were measured using specific ELISA kits (BIOTEND, China) according to the manufacturers’ instructions.

### 2.6 Cytokines screening experiment

Human primary fibroblasts were stimulated with recombinant human TNFα (50 ng/mL), IFNγ (40 ng/mL), IL4 (20 ng/mL), IL13 (20 ng/mL), IL17A (20 ng/mL), IL22 (20 ng/mL), TGFβ1 (5 ng/mL), CXCL1 (40 ng/mL), CXCL5 (40 ng/mL), IL1α (20 ng/mL), or IL1β (20 ng/mL) for 24 h. Cells were lysed directly in TRIzol for RNA extraction, and *MFGE8* expression was measured by RT-qPCR. Detailed information on cytokine sources and catalog numbers is provided in Supplementary Table 3.

### 2.7 Transwell migration assay

NIH/3T3 cells were pretreated with recombinant MFGE8 (1 μg/ mL; R&D Systems, United States) for 24 h prior to the assay. Subsequently, 5 × 10^4^ RAW264.7 cells suspended in 100 μL DMEM medium were seeded into the upper chamber of Transwell inserts (Corning, United States). A total of 5 × 10^5^ MFGE8-pretreated NIH/3T3 cells in 500 μL DMEM medium were added to the lower chamber. The transwell plates were incubated at 37 °C for 24 h. Cells remaining on the upper surface of the membrane were carefully removed, whereas migrated cells on the lower surface were fixed with 4% paraformaldehyde for 25 min and stained with 0.1% crystal violet (Beyotime, China) for 15 min. Four randomly selected fields were imaged using a light microscope, and the number of migrated cells was quantified with ImageJ 1.49 software (National Institutes of Health, United States; available at https://imagej.net/).

### 2.8 Mice treatment

Genetically diabetic male mice (*db/db*; BKS. Cg- + Leprdb/+Leprdb) and C57BL/6 WT mice were purchased from Cyagen Biosciences (Suzhou, China). Mice were maintained in the Institute of Experimental Animal Research of the Hospital of Dermatology, Chinese Academy of Medical Sciences under specific pathogen-free conditions. The wound modeling procedure in mice was performed as previously described (Li et al., 2024). Briefly, full-thickness excisional wounds (6 mm in diameter, extending through the panniculus carnosus) were created on the dorsal skin of eight-week-old mice using a biopsy punch. Mice were housed individually throughout the experimental period. For analysis of MFGE8 expression, wound tissues were collected on postoperative day 3 (POD3) for RNA extraction or immunofluorescence staining. To evaluate the therapeutic effect of MFGE8-pretreated fibroblasts on diabetic wound healing, mice were given a single injection at the wound edge on the day of wounding with one of three treatments: 100 µL PBS, 100 µL PBS containing PBS-pretreated NIH/3T3 cells (1 × 10^7^ cells/mL), or 100 µL PBS containing NIH/3T3 cells (1 × 10^7^ cells/mL) pretreated with 1 μg/mL MFGE8 protein for 24 h (R&D Systems, United States). Wound areas were photographed every other day until approximately 90% closure was reached in one of the experimental groups. At each time point, a silicone ring with an inner diameter of 10 mm was temporarily placed around the lesion as a scale reference. The area was quantified using ImageJ 1.49 software (National Institutes of Health, United States; available at https://imagej.net/). Tissues were collected on POD6 and POD10 for subsequent analyses.

### 2.9 Modified Masson’s Trichrome Stain Kit

Paraffin sections were deparaffinized and rehydrated, followed by staining with a Modified Masson’s Trichrome Stain Kit (Solarbio, China) in accordance with the manufacturer’s instructions. After mounting with neutral balsam (Solarbio, China), the stained sections were examined under a microscope (Leica Microsystems, Germany).

### 2.10 Bulk RNA sequencing and data analysis

Wound tissues were collected from *db/db* mice on POD6 in the PBS and MFGE8-pretreated fibroblast groups. Total RNA was extracted from these wound tissues using TRIzol Reagent, with three biological replicates per group (n = 3/group). RNA concentration and quality were assessed with a NanoDrop spectrophotometer. Three micrograms of total RNA were used for library preparation. Poly(A)+ mRNA was enriched with poly-T oligo–attached magnetic beads, fragmented, and reverse-transcribed to generate first- and second-strand cDNA. After end repair, A-tailing, and adaptor ligation, cDNA fragments of 400–500 bp were selected using the AMPure XP system (Beckman Coulter, United States). Libraries were amplified by 15 cycles of PCR, purified, and quantified using an Agilent Bioanalyzer 2100. Sequencing was performed on the NovaSeq 6000 platform (Illumina) by Shanghai Personal Biotechnology Co., Ltd. Differentially expressed genes (DEGs) were defined as those genes with |log2 fold change| ≥ 0.3 and P value <0.05. Functional enrichment analyses, including Gene Ontology, Wiki-Pathways, and transcription factor enrichment analysis, were conducted using an online tool (https://maayanlab.cloud/Enrichr/). Heatmap was generated for upregulated inflammatory genes using the “pheatmap” R package with the same cutoff criteria. Lollipop plot was generated using the “ggplot2” package in R to visualize differentially expressed canonical macrophage polarization-associated markers in the MFGE8-Fb group relative to the PBS group. Group differences were calculated as log2 fold-change based on log2(FPKM+1) values, and genes with P value <0.05 were included in the plot.

### 2.11 Analyses of public bulk and scRNA-seq data

Bulk RNA-seq data from GSE199939 (human foot normal skin vs. diabetic foot ulcer tissue) were analyzed in RStudio (v4.4.2) to compare MFGE8 expression abundance between the DFU and control groups, and results were visualized using violin plots.

For scRNA-seq, datasets were obtained from GSE165816 (DFU) and GSE265972 (venous leg ulcer, VLU). The DFU scRNA-seq dataset comprised four clinical conditions: healthy non-diabetic subjects, diabetic subjects without DFU, patients with healing DFU (DFU_H), and patients with non-healing DFU (DFU_NH). Our primary analyses focused on healthy non-diabetic controls, DFU_H, and DFU_NH, assessing MFGE8 expression across all cell subpopulations within each condition. In addition, we evaluated the expression patterns of multiple inflammatory mediators specifically within fibroblasts and macrophages.

The VLU scRNA-seq dataset included normal lower-extremity skin and venous ulcer lesions, and we primarily compared MFGE8 expression across all annotated cell populations between these two conditions. Both scRNA-seq datasets were processed in RStudio (v4.4.2) using the same analytical workflow and quality-control standards as described in the referenced article (Wang J. et al., 2025).

### 2.12 Statistical analysis

Statistical analyses were performed using GraphPad Prism 8 (GraphPad Software, United States), and data are presented as mean ± standard deviation (SD). Comparisons between two groups were performed using Student’s t-test, while comparisons among multiple groups were conducted with one-way ANOVA. For experiments involving multiple groups measured across different time points, two-way ANOVA was applied.

## 3 Results

### 3.1 MFGE8 is reduced in fibroblasts of diabetic wounds

**FIGURE 1:**
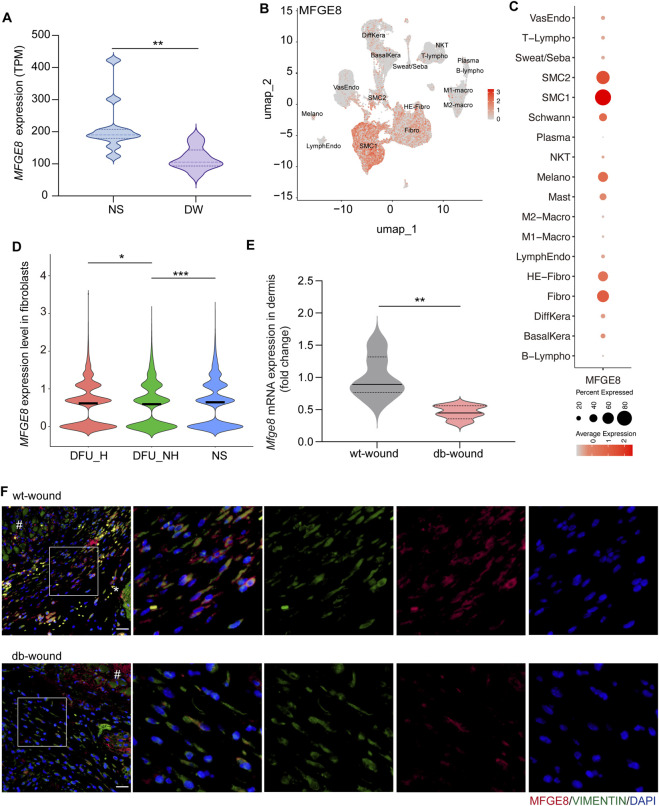
MFGE8 expression is decreased in the fibroblasts of diabetic wounds. **(A)** Violin plot showing *MFGE8* transcript abundance (TPM) in human normal skin (NS) and diabetic wound (DW) samples from the RNA-seq dataset (GSE199939). **(B)** UMAP plot of *MFGE8* expression in scRNA-seq profiling of human diabetic foot ulcers (GSE165816). BasalKera: basal keratinocytes; DiffKera: differentiated keratinocytes; Melano: melanocytes; Fibro: fibroblasts; HE-Fibro: healing enriched-fibroblasts; SMC1/2: smooth muscle cells; VasEndo: vascular endothelial cells; LymphEndo: lymphatic endothelial cells; Schwann: Schwann cells; Sweat/Seba: sweat and sebaceous gland cells; Mast: mast cells; M1/2-Macro: macrophages; T-lympho: T lymphocytes; NKT: natural killer T cells; B-lympho: B-lymphocytes; Plasma: plasma cells. **(C)** Dot plot showing *MFGE8* expression in global cell types (GSE165816). **(D)** Violin plot showing MFGE8 expression in fibroblasts of human normal skin, non-healing diabetic foot ulcer and healing diabetic foot ulcer. NS: normal skin; DFU_NH: non-healing diabetic foot ulcer; DFU_H: healing diabetic foot ulcer. **(E)** RT–qPCR analysis of *Mfge8* expression in the dermis of POD3 wound tissue from C57BL/6 wild-type (n = 6) and *db/db* mice (n = 5). POD3: postoperative day 3. **(F)** Immunofluorescence staining of MFGE8 and Vimentin in POD3 wound tissue from C57BL/6 wild-type (wt) and *db/db* (db) mice. * indicates nonspecific staining of red blood cells; # indicates nonspecific staining of scab or necrotic debris. Scale bar, 20 μm. *P < 0.05, **P < 0.01, ***P < 0.001, by two-tailed Student’s t test **(A,E)** or One-way ANOVA analysis **(D)**.

To investigate the expression pattern of MFGE8 in diabetic wounds, we first analyzed a publicly available RNA-sequencing dataset (GSE199939) (Jiang et al., 2024). Transcriptomic profiling revealed a significant downregulation of *MFGE8* mRNA in full-thickness tissue samples from DFUs compared with healthy skin controls, indicating impaired MFGE8 expression at the tissue level in human diabetic wounds (Figure 1A). To investigate the cellular expression of *MFGE*8 in diabetic wounds, we further analyzed the single-cell RNA sequencing data from human DFUs (GSE165816). We found that *MFGE8* was primarily expressed in structural cells, including fibroblasts and smooth muscle cells (Figures 1B,C). Considering the increasing recognition of the immune regulatory role of fibroblasts in skin wound healing (Theocharidis et al., 2022; Wang J. et al., 2025), we focused on the function of MFGE8 in fibroblasts. Our analysis revealed that in the non-healing group of diabetic foot ulcers, *MFGE8* expression was downregulated compared to normal skin, and it showed partial recovery in the healing group (Figure 1D). Additionally, in venous leg ulcers, *MFGE8* was mainly expressed in fibroblasts and smooth muscle cells (Supplementary Figure S1A), with a reduction in the fibroblasts of the disease group (Supplementary Figure S1B). These data suggest that low MFGE8 expression in fibroblasts may be a common feature of chronic wounds.

To determine whether this reduction is specific to the chronic wound environment, we created full-thickness excisional wounds in wild-type and *db/db* mice and analyzed MFGE8 expression in the dermis on postoperative day 3 (POD3). We found that, compared to the normal acute wound healing process in wild-type mice, *Mfge8* mRNA levels were downregulated in the dermis of *db/db* mice (Figure 1E). Immunostaining also revealed a decrease of MFGE8-positive fibroblasts in the wound of diabetic mice (Figure 1F).

Taken together, these findings suggest that diabetic wounds fail to maintain MFGE8 in the dermis, suggesting impaired upregulation of MFGE8 in fibroblasts potentially contributing to delayed healing in diabetic skin.

### 3.2 MFGE8 drives fibroblasts into a pro-inflammatory state

**FIGURE 2:**
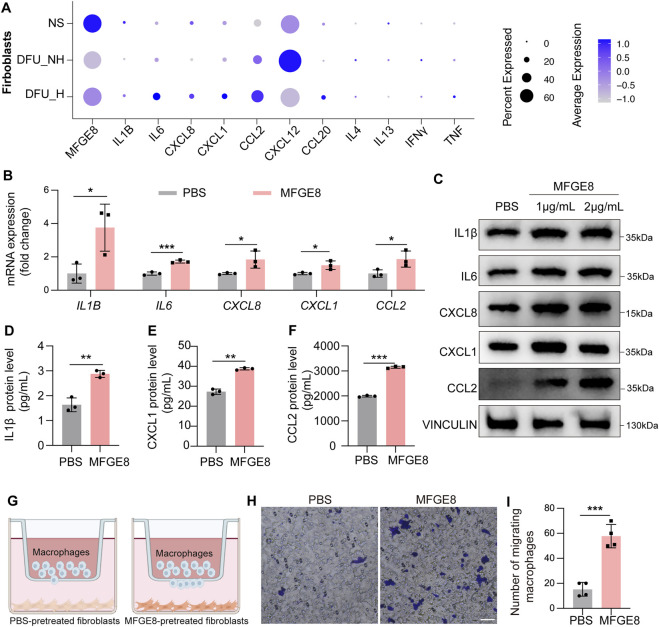
MFGE8 promotes inflammatory response of fibroblasts. **(A)** Dot plot showing *MFGE8* and several inflammatory cytokines expression in fibroblasts of human normal skin, non-healing diabetic foot ulcer and healing diabetic foot ulcer (GSE165816). **(B)** RT–qPCR analysis of *IL1B*, *IL6*, *CXCL8*, *CXCL1* and *CCL2* in PBS-treated and MFGE8-treated human primary fibroblasts (n = 3/group). **(C)** Western blot analysis of inflammatory cytokines expression in PBS-treated and MFGE8-treated HPFs. **(D–F)** ELISA analysis of the protein levels of IL6, CXCL1, and CCL2 in the cell culture supernatants of PBS-treated and MFGE8-treated HPFs (n = 3/group). **(G)** Schematic diagram showing the use of PBS-pretreated and MFGE8-pretreated NIH/3T3 cell to induce RAW264.7 cell migration. **(H)** RAW264.7 cell migration induced by PBS-pretreated and MFGE8-pretreated NIH/3T3 cell (n = 4/group). Scale bar, 50 μm. **(I)** Quantitative analysis of RAW264.7 cell migration induced by PBS-pretreated and MFGE8-pretreated NIH/3T3 cell. *P < 0.05, **P < 0.01, ***P < 0.001, by two-tailed Student’s t-test **(B, D–F, I)**.

To investigate whether MFGE8 promotes chronic wound healing by shaping the inflammatory phenotype of fibroblasts, we first profiled the expression of key inflammatory factors known to change during cutaneous wound repair (Theocharidis et al., 2022; Peña et al., 2024) across normal skin and diabetic wound samples from the healing and non-healing groups. Compared with normal skin, *IL1B*, *CXCL1*, *CXCL8*, and *CCL20* were downregulated in fibroblasts from non-healing diabetic wounds, accompanied by reduced *MFGE8* expression. In contrast, fibroblasts from healing diabetic wounds exhibited increased expression of *IL1B*, *IL6*, *CXCL8*, *CXCL1*, *CCL2*, *CCL20*, and *TNF* relative to non-healing diabetic wounds, together with a restoration of *MFGE8* expression (Figure 2A). These findings suggest that fibroblasts in diabetic wounds—particularly in non-healing lesions—display a blunted, low-grade inflammatory transcriptional program, and that the expression patterns of these inflammatory factors parallel MFGE8 levels in diabetic wounds.

To directly test whether MFGE8 can drive inflammatory cytokine production in fibroblasts, we treated human primary fibroblasts (HPFs) with recombinant MFGE8 protein and assessed cytokine/chemokine expression. MFGE8 stimulation robustly increased the expression of the classic pro-inflammatory cytokines IL1β and IL6, as well as the monocyte/macrophage-recruiting chemokine CCL2 and the neutrophil-attracting chemokines CXCL1 and CXCL8, at both the mRNA and protein levels (Figures 2B,C). However, MFGE8 had no effect on the mRNA expression of *IL4*, *IL13*, *TNF*, or *CXCL12* in fibroblasts (Supplementary Figures S2A–D). Consistent with these findings, MFGE8-treated fibroblasts secreted significantly higher levels of IL1β, CXCL1, and CCL2 into the culture supernatant than PBS control (Figures 2D–F). Notably, CCL2 concentrations reached 3,000 pg/mL in the MFGE8-treated group, markedly exceeding the levels of IL1β and CXCL1 detected in the supernatant (Figures 2D–F). Given that CCL2 is a potent chemoattractant for monocytes/macrophages, we hypothesized that MFGE8-pretreated fibroblasts may promote macrophage recruitment via a CCL2-dependent paracrine mechanism. Transwell migration assays showed that the MFGE8-pretreated fibroblast group significantly promoted macrophage migration across the membrane compared with the PBS-pretreated group (Figures 2G–I).

Movere, the transcript levels of *COL1A1*, *ACTA2*, *MMP1*, and *MMP3* were not significantly altered by MFGE8 treatment (Supplementary Figures S3A–D), suggesting that MFGE8 has minimal impact on canonical fibroblast functions related to extracellular matrix (ECM) production and remodeling.

Taken together, these data indicate that MFGE8 drives dermal fibroblasts toward a pro-inflammatory and chemokine-producing state that enhances macrophage recruitment, without directly perturbing classical ECM-related programs.

### 3.3 TNFα and IL1α promote MFGE8 expression in skin fibroblasts

**FIGURE 3:**
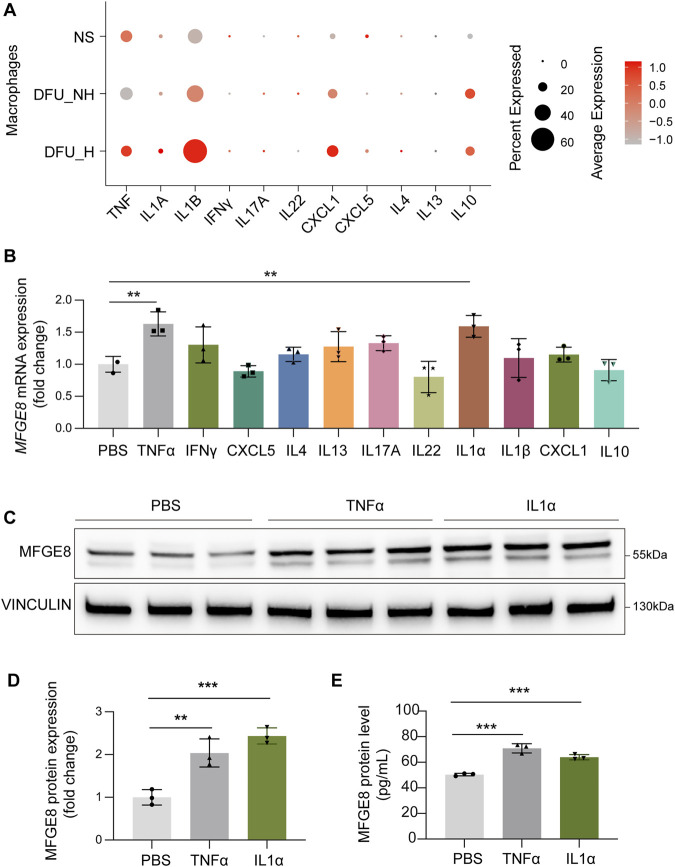
TNFα and IL1α promote MFGE8 expression in skin fibroblasts. **(A)** Dot plot showing several inflammatory cytokines expression in macrophages of human normal skin, non-healing diabetic foot ulcer and healing diabetic foot ulcer (GSE165816). **(B)** RT-qPCR analysis of *MFGE8* expression in HPFs treated with a series of cytokines (n = 3/group). **(C)** Western blot analysis of MFGE8 expression in HPFs treated with TNFα, IL1α or PBS (n = 3/group). **(D)** Quantitative analysis of MFGE8 protein levels relative to VINCULIN. **(E)** ELISA analysis of the protein levels of MFGE8 in the cell culture supernatants of HPFs treated with TNFα, IL1α or PBS (n = 3/group). **P < 0.01, ***P < 0.001, by one-way ANOVA analysis **(B, D, E)**.

Compared to acute cutaneous and oral wounds, chronic diabetic ulcers exhibit reduced macrophage infiltration and attenuated expression of multiple inflammatory cytokines (Sawaya et al., 2020). Similarly, we analyzed the expression of several classic inflammatory cytokines in macrophages from diabetic wounds and found that, compared to normal skin, *TNF*, *IFNγ*, and *CXCL5* were downregulated in the non-healing DFU group. In contrast, in the healing DFU group, the expression of *TNF*, *IL1A*, *IL1B*, *IFNγ*, *CXCL1*, *CXCL5*, and *IL4* was significantly upregulated (Figure 3A). These findings suggest that macrophages in diabetic wounds are in a state of low-intensity immune activation, and the expression of macrophage-derived inflammatory cytokines follows a similar trend to the expression of MFGE8 in fibroblasts.

We next asked whether diminished inflammatory signaling could underlie the aberrant downregulation of MFGE8 observed in diabetic wounds. To address this, HPFs were stimulated with a panel of cytokines implicated in tissue repair and inflammatory activation. Among the factors tested, TNFα and IL1α emerged as the most potent inducers of MFGE8, producing a marked increase in *MFGE8* mRNA relative to PBS controls (Figure 3B). Consistent with these transcriptional changes, TNFα and IL1α also increased MFGE8 protein levels in fibroblast lysates, indicating that MFGE8 is a highly cytokine-inducible gene in dermal fibroblasts (Figures 3C,D).

Previous studies have established that MFGE8 promotes diabetic wound repair by regulating macrophage polarization and angiogenesis (Uchiyama et al., 2017; Kuang et al., 2023). To further investigate the regulatory effects on MFGE8 secretion, we quantified MFGE8 levels in the culture supernatants of dermal fibroblasts following cytokine stimulation. Both TNFα and IL1α stimulation resulted in a significant increase in MFGE8 secretion, as measured by ELISA of conditioned media (Figure 3E). This suggests that pro-inflammatory signals can amplify fibroblast-secreted MFGE8, enabling its effect on neighboring immune and endothelial cells and potentially reinforcing pro-repair signaling within the diabetic wound microenvironment.

Collectively, these data identify TNFα and IL1α as key upstream regulators of fibroblast MFGE8 expression, and raise the possibility that a deficient cytokine milieu in diabetic wounds contributes to the failure to appropriately upregulate MFGE8 in the dermal compartment.

### 3.4 A single dose of MFGE8-pretreated fibroblasts restores impaired healing in diabetic wounds

Previous studies in murine models of diabetic wound healing have shown that recombinant MFGE8 significantly accelerates wound closure with multiple administrations (Das et al., 2016; Uchiyama et al., 2017). However, this approach is cumbersome and may be difficult to translate into clinical practice. Based on our *in vitro* findings, we investigated whether early intervention with MFGE8-pretreated fibroblasts during the inflammatory phase could enhance wound healing in diabetic mice.

**FIGURE 4:**
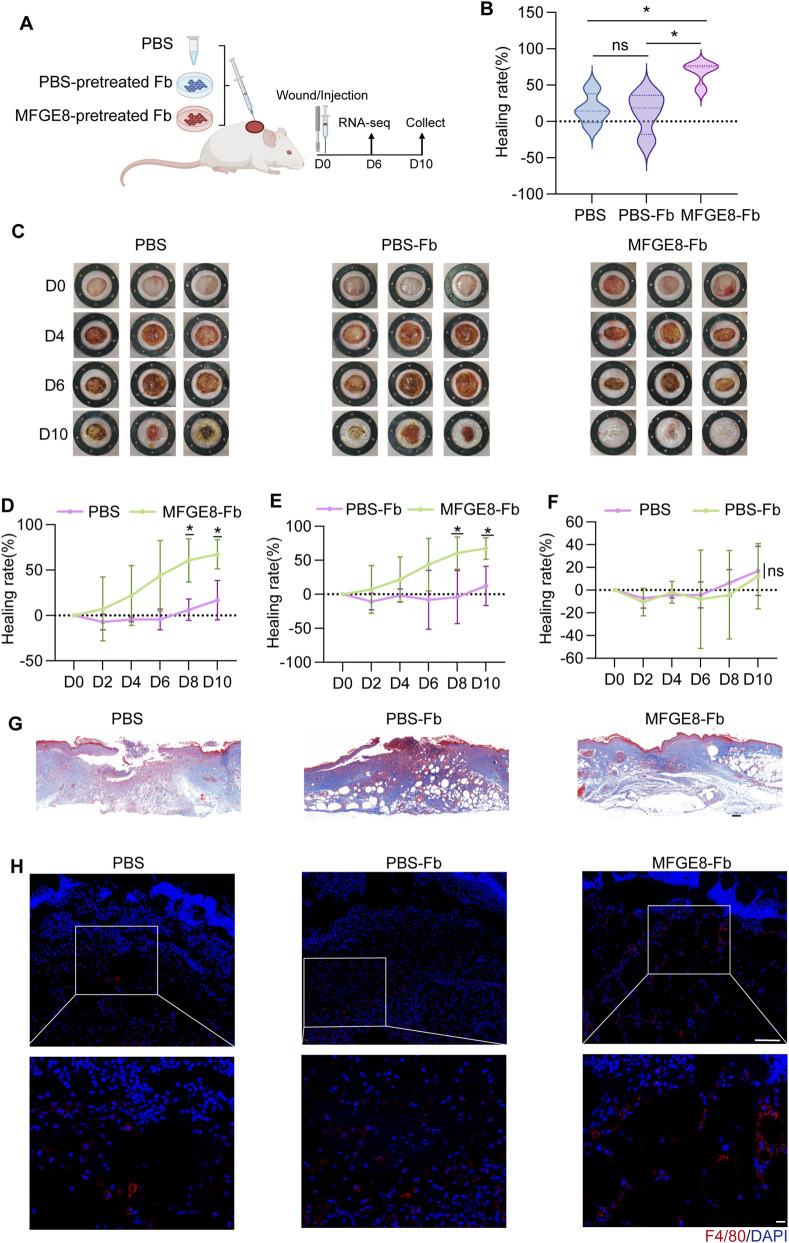
MFGE8-pretreated fibroblasts accelerate diabetic wound healing. **(A)** Schematic diagram showing *db/db* mice received a single administration of recombinant PBS, PBS-pretreated fibroblasts, or MFGE8-pretreated fibroblasts on the day of wounding (n = 4/group). Wound tissues were harvested on day 6 or day 10 for bulk RNA sequencing and subsequent analyses. **(B)** Healing rates across the three experimental groups on day 10 post-wounding. PBS-Fb: PBS-pretreated fibroblasts. MFGE8-Fb: MFGE8-pretreated fibroblasts. **(C)** Representative wound images of the three experimental groups. **(D)** Wound closure rates over time following treatment with PBS versus MFGE8-pretreated fibroblasts. **(E)** Wound closure rates over time following treatment with PBS-pretreated fibroblasts versus MFGE8-pretreated fibroblasts. **(F)** Wound closure rates over time following treatment with PBS-pretreated fibroblasts versus PBS. **(G)** Modified Masson’s Trichrome Staining of wound tissues on day 10 post-wounding. Scale bar, 200 μm. **(H)** Immunofluorescence staining of F4/80 of wound tissues on day 6 post-wounding. Scale bar, 100 μm *P < 0.05, by One-way ANOVA analysis **(B)** or Two-way ANOVA analysis **(D–F)**.

Full-thickness excisional wounds were created in *db/db* diabetic mice and treated once at the time of wounding with one of three interventions: PBS, PBS-pretreated fibroblasts (PBS-Fb), or MFGE8-pretreated fibroblasts (MFGE8-Fb) (Figure 4A). Quantitative analysis of wound area on day 10 showed that MFGE8-Fb group demonstrated significantly greater wound closure rate compared to PBS and PBS-Fb group (Figure 4B). Time-course analysis further elucidated the distinct healing dynamics induced by the MFGE8-based interventions (Figures 4C–E). And there was no significant difference between PBS and PBS-Fb group in wound healing rate (Figures 4C,F). Histological evaluation using Masson’s trichrome staining on day 10 provided further evidence of enhanced repair in the MFGE8-Fb group. Compared to the PBS and PBS-Fb groups, wounds in the MFGE8-Fb group exhibited more complete re-epithelialization, markedly reduced scab and necrotic tissue, and better-organized collagen deposition (Figure 4G). Most importantly, MFGE8-Fb group showed enhanced macrophage infiltration compared with controls (Figure 4H).

These findings demonstrate that a single administration of MFGE8-Fb during the inflammatory phase is sufficient to promote wound healing in a murine diabetic model, highlighting the crucial role of fibroblast-mediated inflammatory regulation in diabetic wound repair.

### 3.5 MFGE8-pretreated fibroblasts activate inflammatory and pro-repair transcriptomic programs in diabetic wounds

To further define the transcriptional programs activated by MFGE8-pretreated fibroblasts *in vivo*, we performed bulk RNA-seq on POD6 wound tissues from *db/db* mice treated with either PBS or MFGE8-pretreated fibroblasts.

**FIGURE 5:**
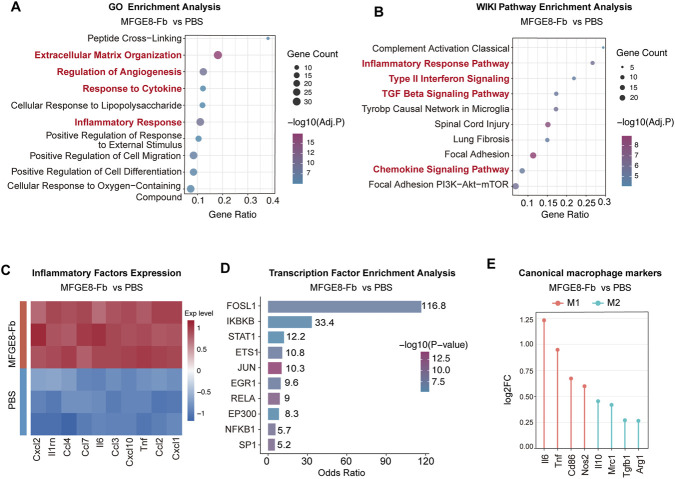
MFGE8-pretreated fibroblasts promote diabetic wound healing through modulation of inflammation, angiogenesis, and ECM synthesis. **(A)** GO enrichment analysis of upregulated DEGs in MFGE8-Fb group compared with PBS control. DEGs: differentially-expressed genes. **(B)** Wiki pathways analysis of upregulated DEGs in MFGE8-Fb group compared with PBS control. **(C)** Heatmap analysis of the expression of inflammatory factors expression in MFGE8-Fb group compared with PBS control. **(D)** Transcription factor enrichment analysis of DEGs in MFGE8-Fb group compared with PBS control. **(E)** Lollipop plot showing the differential expression of canonical macrophage polarization-associated markers in the MFGE8-Fb group relative to the PBS group. Genes were ranked by/log2FC/to highlight the most strongly altered markers. Red labels indicate representative M1-associated genes, and blue labels indicate representative M2-associated genes.

Compared to PBS-treated wounds, the MFGE8-Fb group exhibited significant pathway alterations. Gene Ontology (GO) enrichment analysis revealed significant enrichment of biological processes associated with extracellular matrix organization, angiogenesis, and inflammatory responses (Figure 5A). Consistent with these findings, Wiki-Pathways analysis highlighted the enrichment of type II interferon signaling, chemokine signaling, and TGF-β signaling pathways in the MFGE8-Fb group (Figure 5B). Heatmap analysis further demonstrated the upregulation of multiple inflammatory cytokines and chemokines, including *Il6*, *Tnf*, *Il1rn*, *Cxcl1*, *Cxcl12*, *Ccl2*, *Ccl3*, *Ccl4*, *Ccl7*, and *Cxcl10* in the MFGE8-Fb group (Figure 5C). The transcription factor analysis highlighted the involvement of components associated with the NF-κB, STAT1, and AP-1 complexes, suggesting their potential involvement in the regulatory pathways modulated by MFGE8-pretreated fibroblasts (Figure 5D). Moreover, compared with the PBS group, the MFGE8-Fb group exhibited increased expression of several M1-associated genes (*Il6*, *Tnf*, *Cd86*, and *Nos2*), together with changes in selected M2-associated genes (*Mrc1* and *Il10*), indicating tissue-level changes in macrophage-associated inflammatory and immunoregulatory transcriptional programs, with a predominantly pro-inflammatory signature (Figure 5E).

Collectively, these transcriptomic analyses indicate that MFGE8-pretreated fibroblasts promote diabetic wound repair *in vivo* by orchestrating inflammatory, angiogenic, and extracellular matrix remodeling programs.

## 4 Discussion

Chronic diabetic ulcers are characterized by failed tissue repair, accompanied by a less-effective inflammatory response and depletion or dysfunction of healing-competent stromal and immune cell populations (Sawaya et al., 2020; Theocharidis et al., 2020; Theocharidis et al., 2022; Liu Z. et al., 2025). The role of mesenchymal cells as critical coordinators of immune cell activity in both steady state and following injury is increasingly recognized (Krausgruber et al., 2020; Zhou et al., 2022). Among these, fibroblasts serve as potent regulators of early-stage inflammation in wound healing, upregulating various factors, including CCL2, CCL7, and IL33, that are essential for promoting effective wound repair (Almet et al., 2025; Amuso et al., 2025).

Our study identifies MFGE8 as a previously underappreciated regulator of fibroblast inflammatory competence in diabetic wounds. We demonstrate that MFGE8 expression is markedly reduced in fibroblasts from chronic diabetic ulcers. This reduction may contribute to the persistent low-inflammatory or immunosuppressed state characteristic of chronic wounds, thereby impairing the initiation of appropriate immune–stromal crosstalk required for successful repair.

Although prior studies have shown that repeated administration of recombinant MFGE8 accelerates wound closure in diabetic mouse models, protein-based therapy presents inherent limitations in chronic wounds (Das et al., 2016; Uchiyama et al., 2017). The chronic wound microenvironment is protease-rich, with elevated matrix metalloproteinases and other degradative enzymes, and is frequently complicated by persistent bacterial and fungal colonization (Cho et al., 2019; Chen et al., 2023; Ge and Wang, 2022; Legrand and Martino, 2022). Such conditions promote rapid degradation and functional inactivation of recombinant proteins, necessitating repeated dosing and limiting sustained therapeutic efficacy (Legrand and Martino, 2022). Moreover, exogenous protein delivery cannot dynamically respond to local inflammatory cues nor provide coordinated, cell-context–dependent signaling within the evolving wound niche. In contrast, our findings demonstrate that a single local administration of MFGE8-prestimulated fibroblasts markedly accelerates diabetic wound healing. This cell-based strategy offers several key advantages. First, MFGE8-primed fibroblasts function as living therapeutic units capable of sustained cytokine and chemokine production, thereby restoring local inflammatory signaling over time. Second, transplanted cells are inherently more resistant to proteolytic degradation than exogenous recombinant proteins and can adapt to the hostile wound microenvironment. Third, beyond acting as passive carriers of MFGE8, these preconditioned fibroblasts actively reprogram the stromal–immune network, restoring inflammatory competence and facilitating coordinated transition toward tissue repair. Moreover, the cell administration effectively achieving long-lasting repair in DFU with a single treatment, presenting greater clinical applicability. Thus, MFGE8-pretreated fibroblasts function as microenvironment-modifying effectors rather than simple protein delivery vehicles, overcoming fundamental biological barriers that limit protein-based approaches.

Effective skin wound healing relies on complex intercellular communication (Amuso et al., 2025). The formation of a pro-inflammatory environment in the early stages of injury is crucial, characterized by the release of inflammatory cytokines and the recruitment of myeloid cells (Amuso et al., 2025). In our study, recombinant MFGE8 treatment of fibroblasts significantly promoted the synthesis and secretion of pro-inflammatory mediators, including IL1β, CXCL1, and CCL2, with the most notable effect on CCL2 secretion. Moreover, MFGE8-pretreated fibroblasts markedly enhanced macrophage migration. These findings support a model in which MFGE8 restores fibroblast capacity to initiate early inflammatory amplification loops necessary for regenerative healing.

In acute wounds, pro-inflammatory macrophages release cytokines such as TNFα, IL1α/β, CCL2, and CCL7, which target endothelial cells and fibroblasts to promote wound healing (Liu Z. et al., 2025). However, in diabetic foot ulcers, macrophage function is significantly impaired, with marked reductions in gene expression related to cytokine signaling, lipopolysaccharide response, and oxidative stress (Liu Z. et al., 2025). Moreover, in venous ulcers, macrophages tend to polarize toward an anti-inflammatory M2 phenotype, and communication between immune cells and structural cells is diminished (Wang J. et al., 2025). Our *in vitro* experiments show that TNFα and IL1α significantly promote the synthesis and secretion of MFGE8 in fibroblasts. We hypothesize that in diabetic wounds, early macrophage dysfunction or insufficient infiltration results in inadequate inflammatory cytokine signaling, leading to low MFGE8 expression in fibroblasts. This suppression inhibits fibroblasts from transitioning to a chemokine-producing, immune-recruiting phenotype, further decreasing local chemokine secretion and macrophage recruitment, which reinforces a low-immune microenvironment that is unable to effectively transition to the reparative phase.

In summary, the reduced expression of MFGE8 may be a crucial factor contributing to the ineffective inflammatory response observed in chronic diabetic wounds. By restoring MFGE8 expression, it may be possible to reactivate fibroblast inflammatory programs, enhance immune cell recruitment, and accelerate wound healing. Therefore, MFGE8 reprogramming of fibroblast inflammation presents a potential therapeutic target for diabetic wound treatment, offering a promising approach to improving the healing process in chronic wounds.

## 5 Limitation

One limitation of the present study is that MFGE8-induced cytokine responses were characterized in HPFs, whereas the migration assay and *in vivo* cell-transplantation experiments were performed using NIH/3T3 fibroblasts. Therefore, whether NIH/3T3 cells can fully recapitulate the inflammatory response of primary dermal fibroblasts to MFGE8 stimulation, and whether MFGE8-pretreated human primary fibroblasts can likewise promote DFU of human, remain to be further determined. Future studies integrating additional mechanistic investigations and clinically relevant research will be necessary to further validate the translational relevance of our findings. Another limitation of this study is that the RNA-seq analysis compared the PBS group with the MFGE8-Fb group without including the PBS-Fb group. Although no significant difference was observed between the PBS-Fb and PBS groups in either macrophage infiltration or wound healing rate, the observed transcriptomic changes between PBS and MFGE8-Fb cannot fully distinguish the effects of MFGE8 pretreatment from those of fibroblast transplantation itself. Future studies including the Ctrl-Fb group will help clarify the specific contribution of MFGE8 pretreatment.

## Data Availability

The RNA-seq data generated in this study have been deposited in the NCBI Sequence Read Archive (SRA) under BioProject accession number PRJNA1437528.
