# Supplementary material for: MFGE8-primed fibroblasts reprogram the immunosuppressed microenvironment to promote diabetic wound healing
Source: Front Cell Dev Biol. 2026 May 6;14:1810043. doi: 10.3389/fcell.2026.1810043 (PMC13187488; doi:10.3389/fcell.2026.1810043)
Supplement: Supplementary file 1 [file DataSheet1.pdf]

Supplementary Information

Supplementary Figure1.

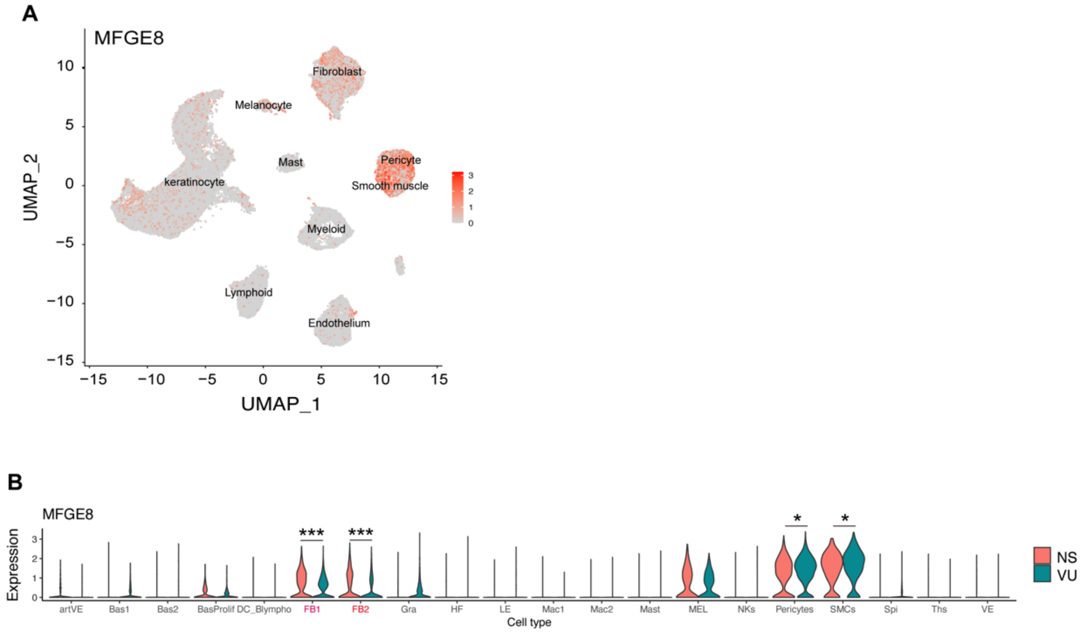

Supplementary figure1. *MFGE8* expression is decreased in the fibroblasts of human venous leg ulcers. (A) UMAP plot of *MFGE8* expression in scRNA-seq profiling of human normal skin (NS) and venous leg ulcers (GSE265972). (B)Violin plot showing *MFGE8* expression in global cell types (GSE265972). *P< 0.05, ***P < 0.001, by Two-way ANOVA analysis (B).

Supplementary Figure2

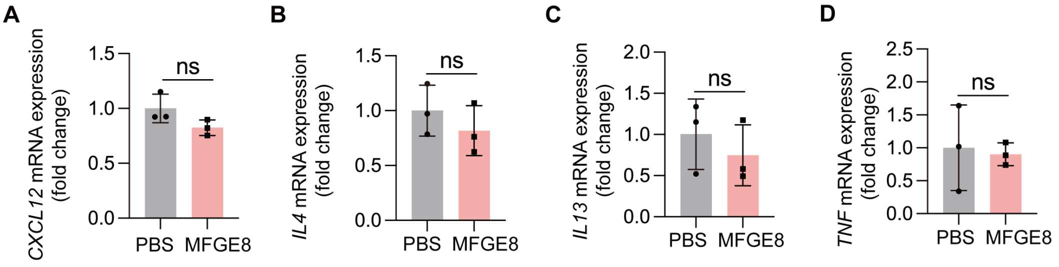

Supplementary figure2. Effects of MFGE8 on several inflammatory cytokine expression in HPFs. (A-D) RT–qPCR analysis of *CXCL12*, *IL4*, *IL13* and *TNFα* in PBS-treated and MFGE8-treated human primary fibroblasts (n=3/group). ns: no significance.

Supplementary Figure3

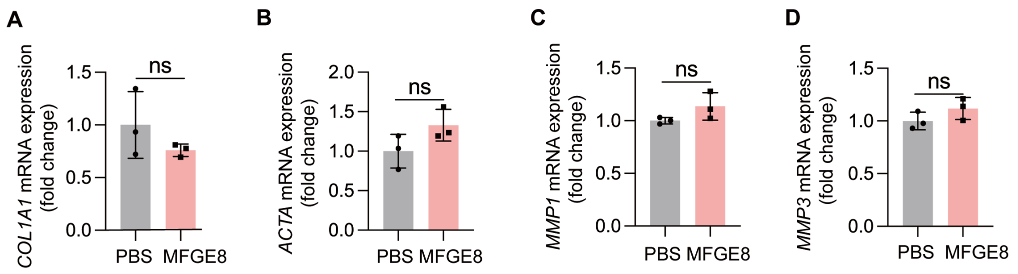

Supplementary figure3. Effects of MFGE8 on several ECM-related gene expression in HPFs. (A-D) RT–qPCR analysis of *COL1A1*, *ACTA2*, *MMP1* and *MMP3* in PBS-treated and MFGE8-treated human primary fibroblasts (n=3/group). ns: no significance.

Supplementary figure4. Graphic abstract.

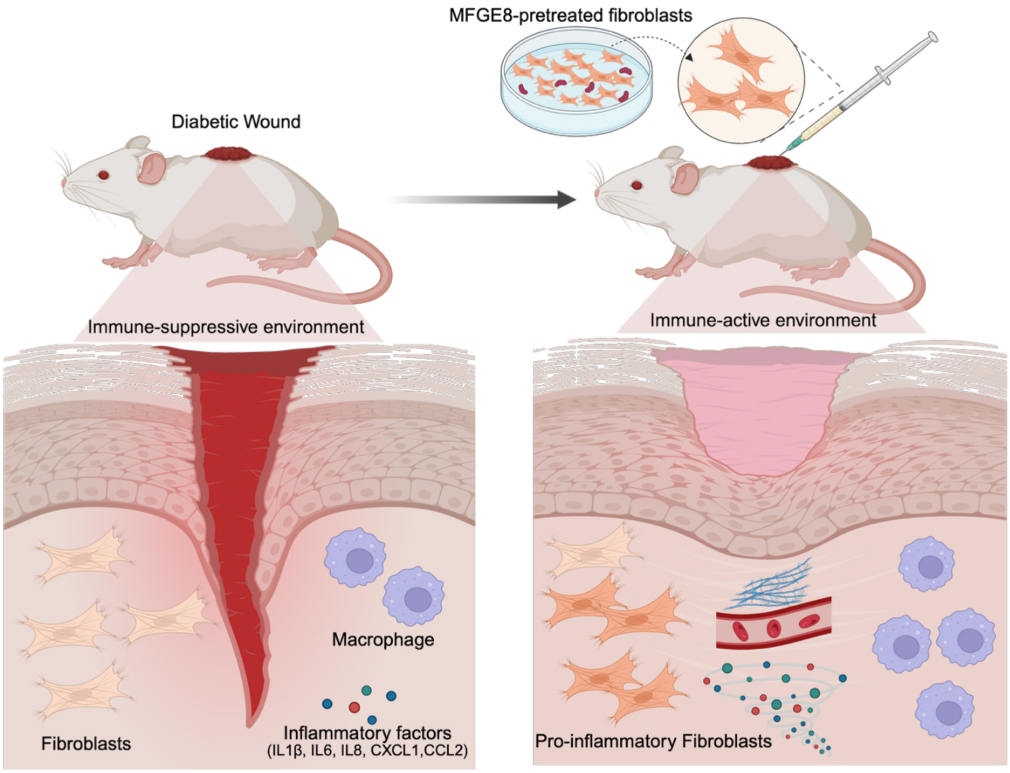

Supplementary Table 1.

RT-qPCR primers	SOURCE	Sequence
MFGE8 (Homo)	Sangon, China	F: CCGTATGAGCAGAGTTCTTCAG<br>R: TGTTCCAGTTACCCACAAACTC
GAPDH (Homo)	Sangon, China	F: GGTGTGAACCATGAGAAGTATGA<br>R: GAGTCCTTCCACGATACCAAAG
COL1A1 (Homo)	Sangon, China	F: AGAGTGGAGAGTACTGGATTGA<br>R: TTCTTGCAGTGGTAGGTGATG
ACTA2 (Homo)	Sangon, China	F: TGTTCCAGCCATCCTTCATC<br>R: CACCGATCCAGACAGAGTATTT
MMP1(Homo)	Sangon, China	F: GCCTTCCAACTCTGGAGTAATG<br>R: GAGCTGCAACACGATGTAAGT
MMP3(Homo)	Sangon, China	F: GGAGATGCCCACTTTGATGA<br>R: AGGTCCATAGAGGGACTGAAT
IL1b(Homo)	Sangon, China	F: GCACCTTCTTTCCCTTCATCT<br>R: ACCACTTGTTGCTCCATATCC
IL6(Homo)	Sangon, China	F: GTAGTGAGGAACAAGCCAGAG<br>R: GGACTGCAGGAACTCCTTAAA
CXCL8(Homo)	Sangon, China	F: GGACAAGAGCCAGGAAGAAA<br>R: GGGTGGAAAGGTTTGGAGTAT
CXCL1 (Homo)	Sangon, China	F: TGCTCCTGCTCCTGGTA<br>R: TGGCTATGACTTCGGTTTGG
CCL2 (Homo)	Sangon, China	F: TCATAGCAGCCACCTTCATTC<br>R: CTCTGCACTGAGATCTTCCTATTG
Mfge8 (Mus)	Sangon, China	F: CCTCGTCTGTGTATATGGGTTTC<br>R: TTGTCTCCACCGCTTTCATC
Gapdh (Mus)	Sangon, China	F: AATGGTGAAGGTCGGTGTG<br>R: GTGGAGTCATACTGGAACATGTAG

Supplementary Table 2.

Antibodies	SOURCE	IDENTIFIER
IL6 antibody	Proteintech, China	26404
MFGE8 antibody	Santacruz, USA	sc-271574
CXCL8 antibody	Huabio, China	ER1901-61
CXCL1 antibody	Huabio, China	HA722841
CCL2 antibody	Huabio, China	EM1710-22
IL1β antibody	Servicebio, China	GB11113
F4/80 antibody	Servicebio, China	GB113373
VINCULIN antibody	Proteintech, China	66305
VIMENTIN antibody	Proteintech, China	10366
Goat anti-Rabbit IgG (H+L) Secondary Antibody, Alexa Fluor™ Plus 488	Thermo Fisher, USA	A32731
Goat anti-Mouse IgG (H+L) Secondary Antibody, Alexa Fluor™ 555	Thermo Fisher, USA	A-21424
Anti-mouse IgG, HRP-linked Antibody	Thermo Fisher, USA	7076
Anti-rabbit IgG, HRP-linked Antibody	Thermo Fisher, USA	7074

Supplementary Table 3

Protein	SOURCE	IDENTIFIER
Homo-MFGE8	Sino Biological, USA	10853-H08B
Mus-MFGE8	Bio-techne, USA	AF-2805
Homo-TNFα	Bio-techne, USA	210-TA
Homo-IL1α	Novoprotein, China	C070
Homo-IL1β	Bio-techne, USA	201-LB
Homo-IFNγ	Bio-techne, USA	285-IF
Homo-IL17A	Bio-techne, USA	317-ILB
Homo-IL22	Novoprotein, China	CH50
Homo-CXCL1	Novoprotein, China	C597
Homo-CXCL5	Novoprotein, China	CF14
Homo-IL4	Bio-techne, USA	204-IL
Homo-IL13	Novoprotein, China	CC89
Homo-IL10	Novoprotein, China	C147
